# PHYSICAL THERAPY STUDENTS' ATTITUDES TOWARDS WORKING WITH PEOPLE WITH DEMENTIA AFTER VARIOUS LEARNING EXPERIENCES

**DOI:** 10.1093/geroni/igz038.3525

**Published:** 2019-11-08

**Authors:** Jennifer L Vincenzo, Holly Bennett

**Affiliations:** University of Arkansas Medical Sciences, Fayetteville, Arkansas, United States

## Abstract

Working with people with dementia (PWD) can be challenging even for the most seasoned health professionals. Hence, teaching health professional students how to effectively work with this patient population is of importance. Two cohorts (n=43; aged 23-36 years) of Graduate Physical Therapy students participated in multi-modal learning experiences geared towards working with PWD within a geriatrics course. Modules included: 1) online lectures and readings followed by a team based learning activity, 2) 3 hours of ‘positive approaches to care’ along with a simulated experience of performing Activities of Daily living and Instrumental Activities of Daily Living of PWD, and 3) one-on-one interactions during both lunch and dinnertime with at least three PWD residing in a state veteran’s home. The Dementia Attitudes Scale (DAS) was used to measure attitudes of students at baseline and following each activity. Repeated measures analysis of variance revealed a significant increase in positive attitudes of students working with PWD across each activity (98.2 +/- 10.5 baseline) with the most positive attitudes noted after interactions with PWD in a state veteran’s home (111.2 +/- 15.0), [F (2.0, 83.8) = 19.4, p < .01, partial eta^2 = .32]. However, this difference was not significant when controlling for students who had previous experience interacting with PWD . In conclusion, Doctor of Physical Therapy students’ attitudes towards PWD improve with different learning experiences, with the greatest improvements after one on one interactions with PWD if the student did not have prior experience interacting with PWD.

